# 2-(4-Chloro­phen­yl)-*N*-(3,4-di­fluoro­phen­yl)acetamide

**DOI:** 10.1107/S1600536813014165

**Published:** 2013-05-31

**Authors:** A. S. Praveen, H. S. Yathirajan, Jerry P. Jasinski, Amanda C. Keeley, B. Narayana, B. K. Sarojini

**Affiliations:** a Department of Studies in Chemistry, University of Mysore, Manasagangotri, Mysore 570 006, India; b Department of Chemistry, Keene State College, 229 Main Street, Keene, NH 03435-2001, USA; c Department of Studies in Chemistry, Mangalore University, Mangalagangotri 574 199, India; d Department of Chemistry, P.A. College of Engineering, Nadupadavu, Mangalore 574 153, India

## Abstract

In the title compound, C_14_H_10_ClF_2_NO, the dihedral angle between the mean planes of the 4-chloro­phenyl and 3,4-di­fluoro­phenyl rings is 65.2 (1)°. These two planes are twisted by 83.5 (5) and 38.9 (9)°, respectively, from that of the acetamide group. In the crystal, N—H⋯O hydrogen bonds form infinite chains along [100]. Weak C—H⋯O and C—H⋯F inter­actions are also observed and stack mol­ecules along the *b* axis.

## Related literature

For the structural similarity of *N*-substituted 2-aryl­acetamides to the lateral chain of natural benzyl­penicillin, see: Mijin & Marinkovic (2006 ▶); Mijin *et al.* (2008 ▶). For the coordination abilities of amides, see: Wu *et al.* (2008 ▶, 2010 ▶). For related structures, see: Praveen *et al.* (2011*a* ▶,*b* ▶,*c* ▶, 2012 ▶). For standard bond lengths, see: Allen *et al.* (1987 ▶).
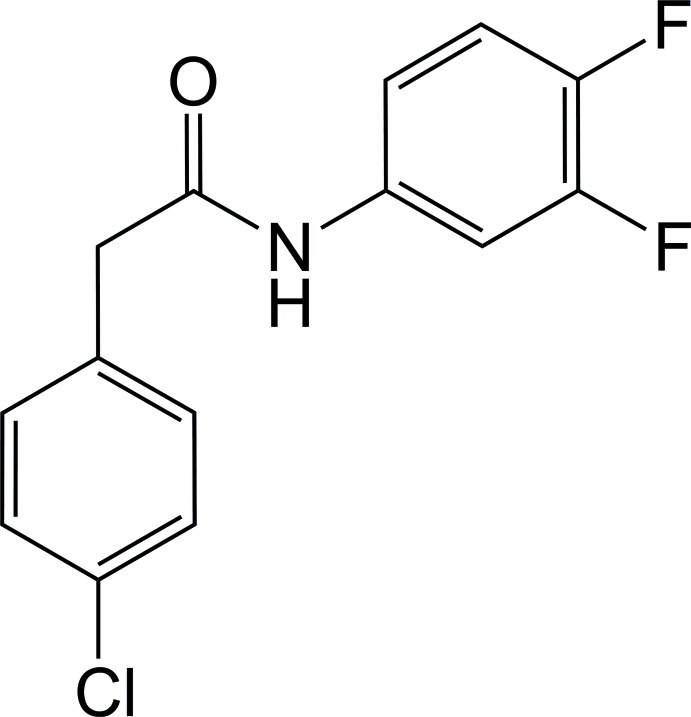


## Experimental

### 

#### Crystal data

C_14_H_10_ClF_2_NO
*M* *_r_* = 281.68
Orthorhombic, 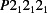

*a* = 4.8935 (5) Å
*b* = 5.8995 (6) Å
*c* = 42.572 (4) Å
*V* = 1229.0 (2) Å^3^
*Z* = 4
Cu *K*α radiation
μ = 2.92 mm^−1^
*T* = 173 K
0.36 × 0.18 × 0.08 mm

#### Data collection

Agilent Xcalibur (Eos, Gemini) diffractometer
Absorption correction: multi-scan (*CrysAlis PRO* and *CrysAlis RED*; Agilent, 2012 ▶) *T*_min_ = 0.608, *T*_max_ = 1.000
7056 measured reflections
2358 independent reflections
2293 reflections with *I* > 2σ(*I*)
*R*_int_ = 0.036

#### Refinement

*R*[*F* ^2^ > 2σ(*F* ^2^)] = 0.042
*wR*(*F* ^2^) = 0.111
*S* = 1.14
2358 reflections
172 parameters
H-atom parameters constrained
Δρ_max_ = 0.42 e Å^−3^
Δρ_min_ = −0.28 e Å^−3^
Absolute structure: Flack x determined using 852 quotients [(I+)−(I−)]/[(I+)+(I−)] (Parsons & Flack, 2004 ▶).
Flack parameter: −0.003 (14)

### 

Data collection: *CrysAlis PRO* (Agilent, 2012 ▶); cell refinement: *CrysAlis PRO*; data reduction: *CrysAlis PRO*; program(s) used to solve structure: *SHELXS97* (Sheldrick, 2008 ▶); program(s) used to refine structure: *SHELXL2012* (Sheldrick, 2008 ▶); molecular graphics: *OLEX2* (Dolomanov *et al.*, 2009 ▶); software used to prepare material for publication: *OLEX2* (Dolomanov *et al.*, 2009 ▶).

## Supplementary Material

Crystal structure: contains datablock(s) global, I. DOI: 10.1107/S1600536813014165/sj5323sup1.cif

Structure factors: contains datablock(s) I. DOI: 10.1107/S1600536813014165/sj5323Isup2.hkl

Supplementary material file. DOI: 10.1107/S1600536813014165/sj5323Isup3.cml

Additional supplementary materials:  crystallographic information; 3D view; checkCIF report

## Figures and Tables

**Table 1:** Hydrogen-bond geometry (Å, °)

*D*—H⋯*A*	*D*—H	H⋯*A*	*D*⋯*A*	*D*—H⋯*A*
N1—H1⋯O1^i^	0.88	1.97	2.854 (4)	177
C5—H5⋯O1^ii^	0.95	2.63	3.307 (4)	129
C14—H14⋯F1^iii^	0.95	2.69	3.615 (5)	164

Symmetry codes: (i) 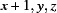; (ii) 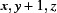; (iii) 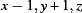.

**Fig. 1.**
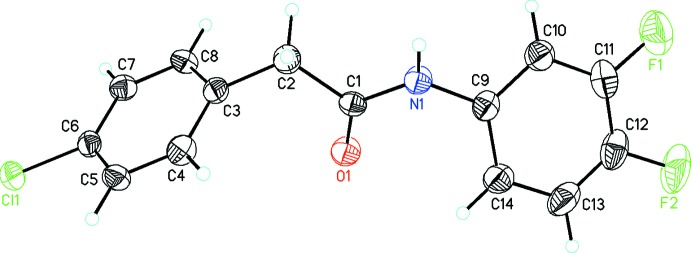
Molecular structure of the title compound showing the atom labeling scheme and 30% probability displacement ellipsoids.

**Fig. 2.**
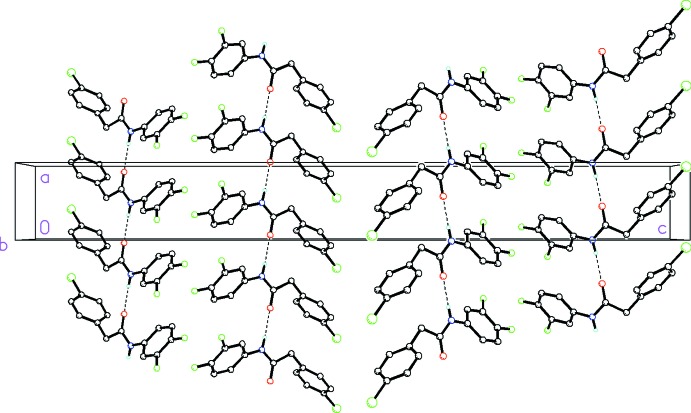
Packing diagram of the title compound viewed along the b axis. Dashed lines indicate N—H···O hydrogen bonds forming infinite chains along (100). H atoms not involved in hydrogen bonding have been deleted for clarity.

**Fig. 3.**
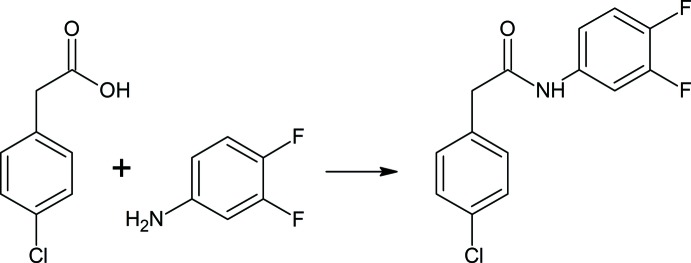
Synthesis of (I).

## supplementary crystallographic information

### Comment

N-Substituted 2-arylacetamides are very interesting compounds because of their structural similarity to the lateral chain of natural benzylpenicillin (Mijin *et al.*, 2006, 2008). Amides are also used as ligands due to their excellent coordination abilities (Wu *et al.*, 2008, 2010). Crystal structures of some acetamide derivatives viz., N-(3-chloro-4-fluorophenyl)-2-(naphthalen-1-yl)acetamide (Praveen *et al.*, 2011*a*), N-(4-chloro-1,3-benzothiazol-2-yl)-2- (3-methylphenyl)acetamide monohydrate (Praveen *et al.*, 2011*b*), N-(3-chloro-4-fluorophenyl)-2,2-diphenylacetamide (Praveen *et al.*, 2011*c*) and N-(4,6-dimethoxypyrimidin-2-yl)-2-(3-methylphenyl)acetamide (Praveen *et al.*, 2012) have been reported. In view of the importance of amides, we report here the crystal structure of the title compound, C_14_H_10_ClF_2_NO, (I).

In (I) the dihedral angle between the mean planes of the 4-chlorophenyl and 3,4-difluorophenyl rings is 65.2 (1)° (Fig. 1). These two planes are twisted by 83.5 (5)° and 38.9 (9)°, respectively, from that of the acetamide group. Bond lengths are in normal ranges (Allen *et al.*, 1987). In the crystal, N—H···O hydrogen bonds are observed forming infinite chains along [100] (Fig. 2). Weak C5–H5···O1 and C14–H14···F1 intermolecular interactions are also observed, Table 1, stacking molecules along the *b* axis and contributing to the packing stability.

### Experimental

4-Chlorophenylacetic acid (0.168 g, 1 mmol), 3,4-difluoro aniline (0.129 g, 1 mmol) and 1-ethyl-3-(3-dimethylaminopropyl)carbodiimide hydrochloride (1.0 g, 0.01 mol) were dissolved in dichloromethane (20 mL). The mixture was stirred in presence of triethylamine at 273 K for about 3 h. The contents were poured into 100 ml of ice-cold aqueous hydrochloric acid with stirring and extracted thrice with dichloromethane. The organic layer was washed with saturated NaHCO_3_ solution and brine solution, dried and concentrated under reduced pressure to give the title compound (I). Single crystals were grown from a dichloromethane and ethyl acetate (1:1) mixture by the slow evaporation method (m.p.: 394–396 K).

### Refinement

All of the H atoms were placed in their calculated positions and then refined using the riding model with atom—H lengths of 0.95Å (CH), 0.99Å (CH_2_) or 0.88° (NH). Isotropic displacement parameters for these atoms were set to 1.2 (CH, CH_2_, NH) times *U*_eq_ of the parent atom.

### Crystal data

**Table:**

C_14_H_10_ClF_2_NO	*D*_x_ = 1.523 Mg m^−^^3^
*M**_r_* = 281.68	Cu *K*α radiation, λ = 1.5418 Å
Orthorhombic, *P*2_1_2_1_2_1_	Cell parameters from 2751 reflections
*a* = 4.8935 (5) Å	θ = 4.2–71.8°
*b* = 5.8995 (6) Å	µ = 2.92 mm^−^^1^
*c* = 42.572 (4) Å	*T* = 173 K
*V* = 1229.0 (2) Å^3^	Block, colourless
*Z* = 4	0.36 × 0.18 × 0.08 mm
*F*(000) = 576	

### Data collection

**Table:**

Agilent Xcalibur (Eos, Gemini) diffractometer	2358 independent reflections
Radiation source: Enhance (Cu) X-ray Source	2293 reflections with *I* > 2σ(*I*)
Graphite monochromator	*R*_int_ = 0.036
Detector resolution: 16.1500 pixels mm^-1^	θ_max_ = 71.9°, θ_min_ = 4.2°
ω scans	*h* = −5→5
Absorption correction: multi-scan (*CrysAlis PRO* and *CrysAlis RED*; Agilent, 2012)	*k* = −5→7
*T*_min_ = 0.608, *T*_max_ = 1.000	*l* = −51→52
7056 measured reflections	

### Refinement

**Table:**

Refinement on *F*^2^	Hydrogen site location: inferred from neighbouring sites
Least-squares matrix: full	H-atom parameters constrained
*R*[*F*^2^ > 2σ(*F*^2^)] = 0.042	*w* = 1/[σ^2^(*F*_o_^2^) + (0.0565*P*)^2^ + 0.5753*P*] where *P* = (*F*_o_^2^ + 2*F*_c_^2^)/3
*wR*(*F*^2^) = 0.111	(Δ/σ)_max_ = 0.001
*S* = 1.14	Δρ_max_ = 0.42 e Å^−^^3^
2358 reflections	Δρ_min_ = −0.28 e Å^−^^3^
172 parameters	Absolute structure: Flack x determined using 852 quotients [(I+)-(I-)]/[(I+)+(I-)] (Parsons & Flack, 2004).
0 restraints	Flack parameter: −0.003 (14)
Primary atom site location: structure-invariant direct methods	

### Special details

**Table:**

Geometry. All esds (except the esd in the dihedral angle between two l.s. planes) are estimated using the full covariance matrix. The cell esds are taken into account individually in the estimation of esds in distances, angles and torsion angles; correlations between esds in cell parameters are only used when they are defined by crystal symmetry. An approximate (isotropic) treatment of cell esds is used for estimating esds involving l.s. planes.

### Fractional atomic coordinates and isotropic or equivalent isotropic displacement parameters (Å^2^)

**Table:**

	*x*	*y*	*z*	*U*_iso_*/*U*_eq_	
Cl1	0.07824 (18)	1.15259 (14)	0.52637 (2)	0.0318 (2)	
F1	1.2311 (7)	−0.3034 (4)	0.69693 (6)	0.0598 (8)	
F2	0.8308 (7)	−0.2387 (5)	0.73854 (6)	0.0667 (9)	
O1	0.5599 (5)	0.4621 (4)	0.62977 (6)	0.0325 (6)	
N1	0.9971 (6)	0.3740 (5)	0.64241 (6)	0.0278 (6)	
H1	1.1690	0.4034	0.6379	0.033*	
C1	0.8043 (7)	0.4794 (5)	0.62509 (7)	0.0235 (7)	
C2	0.9212 (8)	0.6146 (6)	0.59751 (8)	0.0302 (7)	
H2A	1.0618	0.7206	0.6055	0.036*	
H2B	1.0118	0.5088	0.5828	0.036*	
C3	0.7068 (7)	0.7477 (6)	0.57993 (7)	0.0256 (7)	
C4	0.6153 (8)	0.9550 (6)	0.59132 (8)	0.0280 (7)	
H4	0.6866	1.0118	0.6105	0.034*	
C5	0.4220 (8)	1.0801 (5)	0.57515 (7)	0.0274 (7)	
H5	0.3605	1.2217	0.5831	0.033*	
C6	0.3201 (7)	0.9951 (6)	0.54724 (7)	0.0242 (7)	
C7	0.4066 (8)	0.7886 (6)	0.53548 (7)	0.0276 (7)	
H7	0.3341	0.7315	0.5164	0.033*	
C8	0.5996 (7)	0.6671 (6)	0.55194 (7)	0.0281 (7)	
H8	0.6604	0.5254	0.5440	0.034*	
C9	0.9458 (7)	0.2196 (6)	0.66731 (7)	0.0268 (7)	
C10	1.1106 (8)	0.0296 (6)	0.66937 (8)	0.0332 (8)	
H10	1.2496	0.0031	0.6542	0.040*	
C11	1.0702 (9)	−0.1211 (6)	0.69379 (9)	0.0388 (9)	
C12	0.8660 (9)	−0.0846 (7)	0.71526 (9)	0.0414 (10)	
C13	0.7041 (9)	0.1022 (8)	0.71351 (8)	0.0420 (10)	
H13	0.5651	0.1263	0.7287	0.050*	
C14	0.7420 (7)	0.2572 (7)	0.68951 (8)	0.0328 (8)	
H14	0.6298	0.3882	0.6882	0.039*	

### Atomic displacement parameters (Å^2^)

**Table:**

	*U*^11^	*U*^22^	*U*^33^	*U*^12^	*U*^13^	*U*^23^
Cl1	0.0302 (4)	0.0333 (4)	0.0318 (4)	0.0079 (4)	−0.0016 (3)	0.0064 (3)
F1	0.071 (2)	0.0458 (15)	0.0624 (16)	0.0100 (14)	−0.0095 (14)	0.0118 (13)
F2	0.0690 (19)	0.079 (2)	0.0520 (14)	−0.0119 (17)	−0.0029 (13)	0.0405 (14)
O1	0.0169 (13)	0.0455 (14)	0.0351 (12)	−0.0013 (12)	0.0013 (10)	0.0068 (11)
N1	0.0156 (14)	0.0387 (16)	0.0292 (13)	−0.0024 (11)	0.0008 (10)	0.0070 (12)
C1	0.0190 (17)	0.0247 (16)	0.0267 (15)	−0.0005 (13)	0.0014 (12)	−0.0011 (12)
C2	0.0221 (17)	0.0332 (17)	0.0355 (17)	−0.0003 (17)	0.0047 (14)	0.0104 (14)
C3	0.0216 (16)	0.0264 (16)	0.0288 (16)	−0.0002 (14)	0.0052 (13)	0.0072 (13)
C4	0.0282 (19)	0.0296 (16)	0.0263 (15)	−0.0030 (15)	−0.0021 (13)	−0.0010 (13)
C5	0.0298 (18)	0.0225 (15)	0.0298 (15)	0.0022 (15)	0.0040 (14)	−0.0023 (12)
C6	0.0192 (16)	0.0259 (16)	0.0275 (15)	0.0004 (13)	0.0019 (12)	0.0065 (12)
C7	0.0285 (18)	0.0285 (16)	0.0259 (14)	0.0003 (15)	0.0006 (13)	−0.0021 (12)
C8	0.0290 (18)	0.0249 (15)	0.0303 (15)	0.0063 (15)	0.0045 (14)	−0.0014 (12)
C9	0.0210 (16)	0.0343 (18)	0.0251 (14)	−0.0050 (15)	−0.0043 (13)	0.0024 (12)
C10	0.029 (2)	0.0396 (19)	0.0315 (16)	−0.0003 (16)	−0.0016 (14)	0.0014 (14)
C11	0.039 (2)	0.0347 (19)	0.0425 (19)	−0.0027 (19)	−0.0121 (18)	0.0079 (16)
C12	0.036 (2)	0.053 (2)	0.0352 (18)	−0.0142 (18)	−0.0074 (16)	0.0164 (18)
C13	0.033 (2)	0.065 (3)	0.0282 (17)	−0.007 (2)	0.0036 (15)	0.0057 (18)
C14	0.0234 (19)	0.045 (2)	0.0301 (17)	0.0004 (16)	0.0002 (13)	0.0005 (16)

### Geometric parameters (Å, º)

**Table:**

Cl1—C6	1.747 (3)	C5—H5	0.9500
F1—C11	1.339 (5)	C5—C6	1.383 (5)
F2—C12	1.356 (4)	C6—C7	1.383 (5)
O1—C1	1.217 (4)	C7—H7	0.9500
N1—H1	0.8800	C7—C8	1.377 (5)
N1—C1	1.349 (4)	C8—H8	0.9500
N1—C9	1.420 (4)	C9—C10	1.384 (5)
C1—C2	1.530 (4)	C9—C14	1.392 (5)
C2—H2A	0.9900	C10—H10	0.9500
C2—H2B	0.9900	C10—C11	1.382 (5)
C2—C3	1.509 (5)	C11—C12	1.371 (6)
C3—C4	1.390 (5)	C12—C13	1.359 (6)
C3—C8	1.386 (5)	C13—H13	0.9500
C4—H4	0.9500	C13—C14	1.383 (5)
C4—C5	1.383 (5)	C14—H14	0.9500
			
C1—N1—H1	117.3	C6—C7—H7	120.5
C1—N1—C9	125.5 (3)	C8—C7—C6	118.9 (3)
C9—N1—H1	117.3	C8—C7—H7	120.5
O1—C1—N1	124.0 (3)	C3—C8—H8	119.4
O1—C1—C2	122.5 (3)	C7—C8—C3	121.2 (3)
N1—C1—C2	113.5 (3)	C7—C8—H8	119.4
C1—C2—H2A	109.0	C10—C9—N1	117.7 (3)
C1—C2—H2B	109.0	C10—C9—C14	120.2 (3)
H2A—C2—H2B	107.8	C14—C9—N1	122.1 (3)
C3—C2—C1	113.1 (3)	C9—C10—H10	120.5
C3—C2—H2A	109.0	C11—C10—C9	119.0 (4)
C3—C2—H2B	109.0	C11—C10—H10	120.5
C4—C3—C2	120.6 (3)	F1—C11—C10	120.5 (4)
C8—C3—C2	120.7 (3)	F1—C11—C12	119.2 (3)
C8—C3—C4	118.7 (3)	C12—C11—C10	120.3 (4)
C3—C4—H4	119.5	F2—C12—C11	118.3 (4)
C5—C4—C3	121.1 (3)	F2—C12—C13	120.6 (4)
C5—C4—H4	119.5	C13—C12—C11	121.0 (3)
C4—C5—H5	120.6	C12—C13—H13	120.1
C4—C5—C6	118.7 (3)	C12—C13—C14	119.9 (4)
C6—C5—H5	120.6	C14—C13—H13	120.1
C5—C6—Cl1	119.2 (3)	C9—C14—H14	120.3
C5—C6—C7	121.3 (3)	C13—C14—C9	119.5 (4)
C7—C6—Cl1	119.4 (3)	C13—C14—H14	120.3
			
Cl1—C6—C7—C8	179.3 (3)	C4—C5—C6—Cl1	−179.4 (3)
F1—C11—C12—F2	−1.6 (6)	C4—C5—C6—C7	0.3 (5)
F1—C11—C12—C13	177.8 (4)	C5—C6—C7—C8	−0.5 (5)
F2—C12—C13—C14	−179.7 (4)	C6—C7—C8—C3	0.2 (5)
O1—C1—C2—C3	8.0 (5)	C8—C3—C4—C5	−0.3 (5)
N1—C1—C2—C3	−175.1 (3)	C9—N1—C1—O1	3.5 (6)
N1—C9—C10—C11	178.5 (3)	C9—N1—C1—C2	−173.4 (3)
N1—C9—C14—C13	−179.1 (3)	C9—C10—C11—F1	−178.2 (3)
C1—N1—C9—C10	139.2 (4)	C9—C10—C11—C12	1.3 (6)
C1—N1—C9—C14	−42.1 (5)	C10—C9—C14—C13	−0.5 (5)
C1—C2—C3—C4	80.6 (4)	C10—C11—C12—F2	178.9 (4)
C1—C2—C3—C8	−100.1 (4)	C10—C11—C12—C13	−1.6 (6)
C2—C3—C4—C5	179.0 (3)	C11—C12—C13—C14	0.9 (6)
C2—C3—C8—C7	−179.1 (3)	C12—C13—C14—C9	0.2 (6)
C3—C4—C5—C6	0.0 (5)	C14—C9—C10—C11	−0.2 (5)
C4—C3—C8—C7	0.2 (5)		

### Hydrogen-bond geometry (Å, º)

**Table:**

*D*—H···*A*	*D*—H	H···*A*	*D*···*A*	*D*—H···*A*
N1—H1···O1^i^	0.88	1.97	2.854 (4)	177
C5—H5···O1^ii^	0.95	2.63	3.307 (4)	129
C14—H14···F1^iii^	0.95	2.69	3.615 (5)	164

Symmetry codes: (i) *x*+1, *y*, *z*; (ii) *x*, *y*+1, *z*; (iii) *x*−1, *y*+1, *z*.

## References

[bb1] Agilent (2012). *CrysAlis PRO* and *CrysAlis RED* Agilent Technologies, Yarnton, England.

[bb2] Allen, F. H., Kennard, O., Watson, D. G., Brammer, L., Orpen, A. G. & Taylor, R. (1987). *J. Chem. Soc. Perkin Trans. 2*, pp. S1–19.

[bb3] Dolomanov, O. V., Bourhis, L. J., Gildea, R. J., Howard, J. A. K. & Puschmann, H. (2009). *J. Appl. Cryst.* **42**, 339–341.

[bb4] Mijin, D. & Marinkovic, A. (2006). *Synth. Commun.* **36**, 193–198.

[bb5] Mijin, D. Z., Prascevic, M. & Petrovic, S. D. (2008). *J. Serb. Chem. Soc.* **73**, 945–950.

[bb6] Parsons, S. & Flack, H. (2004). *Acta Cryst.* A**60**, s61.

[bb7] Praveen, A. S., Jasinski, J. P., Golen, J. A., Narayana, B. & Yathirajan, H. S. (2011*a*). *Acta Cryst.* E**67**, o1826. 10.1107/S1600536811024597 PMC3151961 21837194

[bb8] Praveen, A. S., Jasinski, J. P., Golen, J. A., Yathirajan, H. S. & Narayana, B. (2011*b*). *Acta Cryst.* E**67**, o2602–o2603. 10.1107/S1600536811035872 PMC3201351 22064942

[bb9] Praveen, A. S., Jasinski, J. P., Golen, J. A., Narayana, B. & Yathirajan, H. S. (2011*c*). *Acta Cryst.* E**67**, o2604. 10.1107/S1600536811036075 PMC3201262 22058752

[bb10] Praveen, A. S., Jasinski, J. P., Golen, J. A., Yathirajan, H. S. & Narayana, B. (2012). *Acta Cryst.* E**68**, o226–o227. 10.1107/S1600536811054493 PMC3254559 22259508

[bb11] Sheldrick, G. M. (2008). *Acta Cryst.* A**64**, 112–122. 10.1107/S0108767307043930 18156677

[bb12] Wu, W.-N., Cheng, F.-X., Yan, L. & Tang, N. (2008). *J. Coord. Chem.* **61**, 2207–2215.

[bb13] Wu, W.-N., Wang, Y., Zhang, A.-Y., Zhao, R.-Q. & Wang, Q.-F. (2010). *Acta Cryst.* E**66**, m288. 10.1107/S160053681000471X PMC2983540 21580233

